# 

**Published:** 1892-09

**Authors:** 


SEPTEMBER, 1892.

*Vol. X.* *No.* **37.**

LIBRARY
Indexed
S. G. O.

### THE BRISTOL MEDICO-CHIRURGICAL JOURNAL

A Quarterly Journal of the Medical Sciences for the West of England and South Wales

*PUBLISHED UNDER THE AUSPICES OF*
*THE BRISTOL MEDICO-CHIRURGICAL SOCIETY.*

"Scire est nescire, nisi id me

Scire alius sciret."

*Editor:* R. SHINGLETON SMITH, M.D., B.Sc.

WITH WHOM ARE ASSOCIATED

BARCLAY J. BARON, M.B., C.M. F. R. CROSS, M.B.
J. MICHELL CLARKE, M.A., M.B. W. H. HARSANT.

*Assistant-Editor:* L. M. GRIFFITHS.

BRISTOL: J. W. ARROWSMITH;
LONDON: J. & A. CHURCHILL, 11 NEW BURLINGTON STREET.

*Price One Shilling and Sixpence.*

